# Sub-millimeter T_2_ weighted fMRI at 7 T: comparison of 3D-GRASE and 2D SE-EPI

**DOI:** 10.3389/fnins.2015.00163

**Published:** 2015-05-05

**Authors:** Valentin G. Kemper, Federico De Martino, An T. Vu, Benedikt A. Poser, David A. Feinberg, Rainer Goebel, Essa Yacoub

**Affiliations:** ^1^Department of Cognitive Neuroscience, Faculty of Psychology and Neuroscience, Maastricht UniversityMaastricht, Netherlands; ^2^Department of Cognitive Neuroscience, Maastricht Brain Imaging Center, Maastricht UniversityMaastricht, Netherlands; ^3^Department of Radiology, Center for Magnetic Resonance Research, University of Minnesota Medical SchoolMinneapolis, MN, USA; ^4^Advanced MRI TechnologiesSebastopol, CA, USA; ^5^Brain Imaging Center, Helen Wills Institute for Neuroscience, University of California, BerkeleyBerkeley, CA, USA; ^6^Department of Neuroimaging and Neuromodeling, Netherlands Institute for Neuroscience, Royal Netherlands Academy of Arts and Sciences (KNAW)Amsterdam, Netherlands

**Keywords:** 3D-GRASE, Spin-Echo EPI, T2, T2^*^, point-spread function, high-resolution BOLD fMRI

## Abstract

Functional magnetic resonance imaging (fMRI) allows studying human brain function non-invasively up to the spatial resolution of cortical columns and layers. Most fMRI acquisitions rely on the blood oxygenation level dependent (BOLD) contrast employing T^*^_2_ weighted 2D multi-slice echo-planar imaging (EPI). At ultra-high magnetic field (i.e., 7 T and above), it has been shown experimentally and by simulation, that T_2_ weighted acquisitions yield a signal that is spatially more specific to the site of neuronal activity at the cost of functional sensitivity. This study compared two T_2_ weighted imaging sequences, inner-volume 3D Gradient-and-Spin-Echo (3D-GRASE) and 2D Spin-Echo EPI (SE-EPI), with evaluation of their imaging point-spread function (PSF), functional specificity, and functional sensitivity at sub-millimeter resolution. Simulations and measurements of the imaging PSF revealed that the strongest anisotropic blurring in 3D-GRASE (along the second phase-encoding direction) was about 60% higher than the strongest anisotropic blurring in 2D SE-EPI (along the phase-encoding direction). In a visual paradigm, the BOLD sensitivity of 3D-GRASE was found to be superior due to its higher temporal signal-to-noise ratio (tSNR). High resolution cortical depth profiles suggested that the contrast mechanisms are similar between the two sequences, however, 2D SE-EPI had a higher surface bias owing to the higher T^*^_2_ contribution of the longer in-plane EPI echo-train for full field of view compared to the reduced field of view of zoomed 3D-GRASE.

## Introduction

Since its discovery more than two decades ago (Bandettini et al., 1992; Kwong et al., 1992; Ogawa et al., 1992), non-invasive functional magnetic resonance imaging (fMRI) has evolved to become a primary method to study human brain function. The majority of fMRI experiments have been conducted using the blood oxygenation level dependent (BOLD) method (Ogawa et al., 1990), relying on T^*^_2_ changes in gradient-echo based echo-planar imaging (GE-EPI, Mansfield, 1977) acquisitions. However, it has been shown in simulations (Boxerman et al., 1995; Uludag et al., 2009; Pflugfelder et al., 2011) and experiments at sub-millimeter (Duong et al., 2003; Yacoub et al., 2003, 2007) or lower resolution (Harmer et al., 2012; Boyacioglu et al., 2014; Panchuelo et al., 2015), that at high field strength (7 Tesla and above) imaging strategies utilizing T_2_ weighted contrast yield higher spatial specificity than T^*^_2_ weighted contrast. This becomes particularly relevant when the investigated functional organization is on the order of 1 mm or smaller, requiring avoidance of pial vein effects that have been shown to be stronger in T^*^_2_ weighted acquisitions (Yacoub et al., 2005). Cerebral blood flow or blood volume based approaches present an alternative approach to high-resolution fMRI (see e.g., Jin and Kim, 2008; Goense et al., 2012; Huber et al., 2015).

By far, most T_2_ weighted fMRI experiments have been conducted using 2D spin-echo EPI sequences (Bandettini et al., 1994; see Norris, 2012 for review). The EPI echo train increases the temporal and the specific absorption rate (SAR) efficiency as compared to purely T_2_ weighted sequences such as RARE/FSE (Hennig et al., 1986; Constable et al., 1994; Poser and Norris, 2007) or to balanced and non-balanced steady-state free precession sequences (b-SSFP/nb-SSFP) (Auerbach et al., 2002; Bieri and Scheffler, 2007; Miller and Jezzard, 2008; Barth et al., 2010; Goa et al., 2014). However, the EPI echo train causes blurring in the phase-encoding direction due to T_2_′ weighting (Constable and Gore, 1992) and increases the T^*^_2_ weighting as demonstrated by Goense and Logothetis (2006).

As an alternative, inner-volume excitation approaches, such as 3D Gradient-and-Spin-Echo (3D-GRASE) (Oshio and Feinberg, 1991; Feinberg et al., 2008) or single slice inner volume 2D-SE EPI (Duong et al., 2002; Yacoub et al., 2003) have been proposed for T_2_ weighted sub-millimeter fMRI in humans. The 3D-GRASE sequence combines EPI-readouts with fast spin-echo acquisition schemes (RARE/TSE) by acquiring multiple EPI readouts separated by refocusing pulses. Several secondary phase-encoding steps (referred to as “partitions” throughout this manuscript) are acquired after a single excitation pulse thus overcoming the single slice limitation of zoomed inner volume EPI. Slab-selective refocusing pulses are applied perpendicularly to the slab-selective excitation pulse to limit the required phase-encoding steps (Feinberg et al., 2008). This inner-volume selection allows for shorter EPI echo-trains, minimizing both T^*^_2_ contrast and blurring in the in-plane phase-encoding direction. At the same time, short EPI echo-trains keep the overall echo train length short to minimize blurring along the partition direction related to T_2_ decay. Centric ordering of the partitions is typically chosen to sample k-space center at maximal SNR (minimal TE). Compared to 2D spin-echo EPI sequences, 3D-GRASE offers a potential SAR advantage because it uses fewer radiofrequency (RF) pulses due to the necessity of only one excitation pulse for the entire 3D volume and a reduced number of refocusing pulses due to partial Fourier acquisitions along the partition direction. The parsimony in SAR can be used to improve the excitation and refocusing slab profiles by increasing the bandwidth-time product of the RF pulses or to shorten the RF pulse duration in order to shorten the overall echo train length. Cortical depth-specific and columnar fMRI studies have been successfully conducted using 3D-GRASE (Zimmermann et al., 2011; Olman et al., 2012), and it has been shown that 3D-GRASE yields a more specific signal than GE-EPI (De Martino et al., 2013). However, thus far, a systematic comparison of 3D-GRASE and 2D SE-EPI has been missing.

Therefore, this study compares 3D-GRASE and 2D SE-EPI for sub-millimeter resolution fMRI experiments at 7 T. We do so by simulating and measuring the imaging point-spread function (referred as to PSF through this manuscript) reflecting the anisotropic spread of information in the image stemming from an idealized point source. The PSF is defined here as the magnitude of the Fourier transform of the complex optical transfer function, a function reflecting a modulation of k-space data (i.e., truncation associated with the finite nominal image resolution and signal reduction by relaxation weighting at different positions in k-space), whose magnitude is the so-called modulation transfer function (MTF) (Robson et al., 1997). T_2_ or T^*^_2_ weighting along an echo-train and truncation of k-space thus impose limits on the effective resolution of the image. Further, we measure temporal stability (temporal signal-to-noise ratio, tSNR), functional responses to visual stimulation, and the cortical depth profiles of the two acquisitions.

## Materials and methods

All measurements were performed on a 7T Siemens Magnetom scanner equipped with a body gradient system (70 mT/m maximum amplitude, 200 mT/m/s maximum slew rate) and a dedicated surface coil (16-channel receive and a separate four channel transmit coil with a pre-determined static B1 phase set optimized for homogeneity over the visual cortex; Live Services, Minneapolis, MN, USA) covering the posterior part of the subjects' heads. Compared to a whole brain coil, this RF coil offers better transmit efficiency (reduced SAR) in occipital areas and a wide angle for visual stimulation. Three subjects [two females, age (28.3 ± 1.5) years] were scanned for imaging PSF and functional measurements. Three additional male subjects (age 33.3 ± 3.5) were scanned for the PSF measurements only (see below). All subjects were scanned after giving informed written consent and in compliance with the local ethical board.

### Visual stimulation protocol

Functional data were acquired while the subjects were performing a visual stimulation task. High contrast concentric flickering checkerboard patterns were displayed centrically on a screen in the magnet bore with a visual angle of 39°. A block design was chosen with 14 blocks of 10 s stimulation and 12 s rest. The subjects were instructed to fixate a point in the middle of the screen.

### Simulations

The signal evolution throughout the echo trains of 3D-GRASE and 2D SE-EPI were simulated in MATLAB (The MATHWORKS Inc., Natick, MA, USA). The same imaging parameters were used in the PSF measurements as in the functional experiments (see below). For the 2D SE-EPI, the signal MTF, (Robson et al., 1997) was modeled as an exponential decay with a time constant (T_2_) of 50 ms (see Uludag et al., 2009 for review of relaxation times) in addition to an exponential decay with a time constant (T_2_′) of 63 ms (where T^*^_2_ = 1/(1/T_2_ + 1/T_2_′) = 27.8 ms) symmetrically on both sides of the central spin-echo (Bernstein et al., 2004), such that the outer k-space lines experience a stronger modulation. To account for partial Fourier acquisitions (Feinberg et al., 1986), the missing k-space lines were zero-filled. Accelerated imaging was accounted for by assuming the acquisition of missing k-space lines interspersed equidistantly between the acquired lines such that the total echo-train duration was maintained.

The in-plane phase-encoding part of the 3D-GRASE sequence was simulated as in the 2D SE-EPI case, whereas the partition direction was simulated to experience an exponential decay with the same time constant (T_2_ = 50 ms, equivalent to perfect 180° refocusing pulses). Centric reordering was taken into account and the same procedure to mimic partial Fourier acquisition was applied.

In the model it was assumed that the modulation along the phase-encoding direction is separable from the modulation along the partition direction in 3D-GRASE. In order to obtain the simulated PSF at a 1 μm resolution the simulated MTF was Fourier transformed after zero-filling to account for the truncated k-space acquisition. This simulation was repeated for the phase-encoding direction of both sequences and the partition direction of 3D-GRASE. All parameter sets used in the experiments were considered. A normal distribution was fitted to the magnitude of each PSF using a linear least squares regression with four free parameters, amplitude, offset, standard deviation, and baseline. A Gaussian was chosen because it well approximated the shape of the central lobe of the PSF (i.e., the convolution of the decay filter and a sinc-kernel due to the k-space truncation) and it allows to easily parameterize the full width at half maximum (FWHM).

### Point-spread function measurements

PSF measurements were performed with the same imaging parameters as the functional measurements (see below) and the simulations, except for the number of measurements and a lower image scaling factor to adjust the dynamic range to accommodate the high image intensity in the k-space center. The vendor's product 2D SE-EPI sequence was used after implementing the option to turn off the phase-encoding gradients. Further, the gradient polarity of the refocussing gradient was inverted to allow low-SAR fat suppression (Park et al., 1987; Volk et al., 1987; Gomori et al., 1988; Ivanov et al., 2010) in the PSF measurements and the functional measurements.

Four volumes were acquired to reach a steady state. In the fifth repetition, the phase-encoding gradients were switched off, so that the k-space center was acquired in every readout line. This procedure yields non-Fourier-encoded images (Fourier-encoded only along the readout direction), which are processed in the image reconstruction pipeline nonetheless. Since the acquired data does not contain any spatial information, but does experience the MTF (i.e., the weighting due T_2_ and T^*^_2_ decay), the resulting image reflects the PSF due to the acquisition and reconstruction (Mugler et al., 1992; Robson et al., 1997; Zaitsev et al., 2004; Park et al., 2007). In the ideal case with no signal decay, all acquisition lines would be identical leading to a reconstructed image with a delta-function like intensity distribution along the non-encoded directions. However, in practice, due to the non-uniformity of the MTF, the peak has a finite width and exhibits side lobes in the case of partial Fourier acquisitions.

The data were analyzed as follows. After standard image reconstruction, individual lines (in phase-encoding or partition encoding direction) were admitted to the analysis if their maximum pixel value was at least 25% of the global image intensity to avoid noise-only lines. These lines were centered on their respective maximums. Then, data were interpolated using a spline interpolation and a normal distribution was fitted as to the simulated data described above. In the 2D SE-EPI acquisitions, as many peaks as the GRAPPA acceleration factor appear in the data due to the aliasing, but only the maximum peak was used for fitting. This makes the results more comparable to the 3D-GRASE data and reduces an overestimation of the PSF due to the collapsing of the PSF over the entire FoV (see Discussion for details).

In order to assess the slice profile of 2D SE-EPI (i.e., the point spread function along the slice direction), we simulated profiles of the used excitation and refocusing pulses by Fourier transforming the vendor-provided RF pulse shapes. Perfect 90 and 180° pulses were assumed at the slice center. The resulting slice profiles were calculated as sin(α_1_) × sin^2^(α_2_/2), where α_1_ and α_2_ denote the spatially dependent excitation and refocusing angles, respectively. The final slice profile was obtained by assuming saturation recovery from the excitation pulse during one TR at the original slice location and during half a TR at the location of the neighboring slices (approximating interleaved slice acquisition). Saturation recovery from both a fully relaxed system (longitudinal magnetization m_z_ = 1) and an Ernst steady state were considered in a range of T_1_ values between 1200 and 1800 ms (step size of 100 ms).

### Functional measurements

Five different acquisitions were repeated two times in each scanning session in a pseudo-random order. All acquisitions had a nominal isotropic resolution of (0.8 mm)^3^ of and repetition time (TR) of 2000 ms. All used nominal excitation/refocussing flip angles of 90/180°. Additional acquisition parameters are listed in Table 1. 3D-GRASE 2 is identical to 3D-GRASE 1 except the image volume was rotated about the read-direction such that the phase-encoding direction of 3D-GRASE 1 was the same as partition encoding direction of 3D-GRASE 2 and vice versa. The slices in all measurements except 3D-GRASE 2 were placed parallel to calcarine sulcus (anterior-posterior, ant-pos), and orthogonally to that in 3D-GRASE 2 (inferior-superior, inf-sup). Phase-encoding direction left-right was chosen for all 2D SE-EPI acquisitions to keep the FoV to a minimum, while avoiding aliasing in the region of interest. Although the echo spacing of the 2D SE-EPI acquisitions had to be slightly increased with respect to 3D-GRASE to avoid critical peripheral nerve stimulation, phase-encoding direction left-right allowed much shorter EPI echo train durations than anterior-posterior in 2D SE-EPI.

**Table 1:** **Acquisition parameters of the five functional imaging protocols**.

	**3D-GRASE 1**	**3D-GRASE 2**	**2D SE-EPI 1**	**2D SE-EPI 2**	**2D SE-EPI 3**
TE (ms)	36	36	36	50	50
Field of view	30 × 120 × 10	30 × 120 × 10	120 × 120 × 16	120 × 120 × 16	120 × 120 × 16
Echo spacing (ms)	1.01	1.01	1.02	1.02	1.03
EPI/total echo train duration (ms)	30/216	30/216	46	46	31
GRAPPA acceleration factor	–	–	2	2	3
Partial Fourier	5/8 (partition direction)	5/8 (partition direction)	6/8 (phase direction)	6/8 (phase direction)	6/8 (phase direction)
Slice orientation	ant-pos	inf-sup	ant-pos	ant-pos	ant-pos

Automatic second order shimming routines were used to homogenize the static magnetic field (B0) in a region of interest big enough to accommodate the 2D SE-EPI FoV. The shim was adjusted manually for further improvements of the homogeneity, until the FWHM of the measured Larmor frequencies was well below 40 Hz. Macroscopic inhomogeneities further cause a reduction in the apparent T^*^_2_. We obtained a macroscopic, volumetric T^*^_2_ of 17 ± 2 ms (mean ± SD across all six participants). The individual subjects' volumetric T^*^_2_ values were also used in the PSF simulations in addition to the theoretical T^*^_2_ values for cortical gray matter. A constant shim was then used throughout all functional and PSF measurements. The transmit field (B1+) was measured using a pre-saturation based B1-mapping sequence provided by the scanner vendor. The transmit voltage was adjusted to yield the correct flip angles in the approximate location of the calcarine sulcus.

### Anatomical measurements

Structural images were acquired for anatomical reference using a T_1_ weighted MP-RAGE sequence (isotropic resolution of 0.6 mm; TR = 3100 ms; TI = 1500 ms; TE = 2.52 ms; flip angle = 5°; GRAPPA acceleration factor = 3; FoV = 230 × 230 × 154 mm^3^). Additional proton density weighted images were acquired using identical imaging parameters (except TR = 1440 ms) without the inversion module. These additional images were used to alleviate inhomogeneities across the FoV in post-processing by dividing the T_1_ weighted image pixel values by those of the proton density weighted images (van de Moortele et al., 2009).

### Functional data analysis

Data analysis was performed using BrainVoyager QX 2.8 (Brain Innovation, Maastricht, The Netherlands) and custom-written routines in MATLAB. Standard preprocessing of the fMRI data consisted of 3D rigid body motion correction and temporal high-pass filtering by a General Linear Model (GLM) with three cosine cycles and a linear trend regressor for each run. The average motion parameters for all acquisitions were 0.32 ± 0.31 mm translation and 0.31 ± 0.28 degree rotation (mean ± SD of absolute deviation of both parameters). No difference between 3D-GRASE and 2D SE-EPI acquisitions was observed. SE-EPI data was additionally slice-time corrected using a sinc-weighted interpolation. These data were then co-registered with the structural images using positional information of the acquisitions. Manual adjustments were performed using edge information in the fMRI data. For this purpose, the fMRI data was averaged across time after preprocessing to yield a single high quality image, with fine-grained structural information (i.e., CSF/gray matter and white matter/gray matter boundaries), for alignment. When image distortions were too strong to guarantee good alignment of the entire volume, multiple alignments were performed specifically in order to optimize the co-registration for the targeted regions, in which cortical depth profiles were generated. Finally, functional images were resampled in the 3D anatomical volume at a resolution of 0.8 mm using a sinc interpolation.

A standard GLM was used to assess the functional activation. The visual stimulation predictor was created by convolving the visual activation blocks with a standard hemodynamic response function (HRF). For each imaging sequence, both runs were analyzed together on a single subject level without additional confound predictors.

tSNR maps were created by dividing the voxelwise temporal mean intensity by the temporal standard deviation. To produce tSNR maps, preprocessing included only high-pass filtering, using four cosine cycles and linear trend removal. No motion correction or slice-time correction was applied to avoid additional spatial interpolation. The effect of the visual stimulation was removed from the voxels' time course using univariate regression (i.e., GLM).

**Figure 1:**
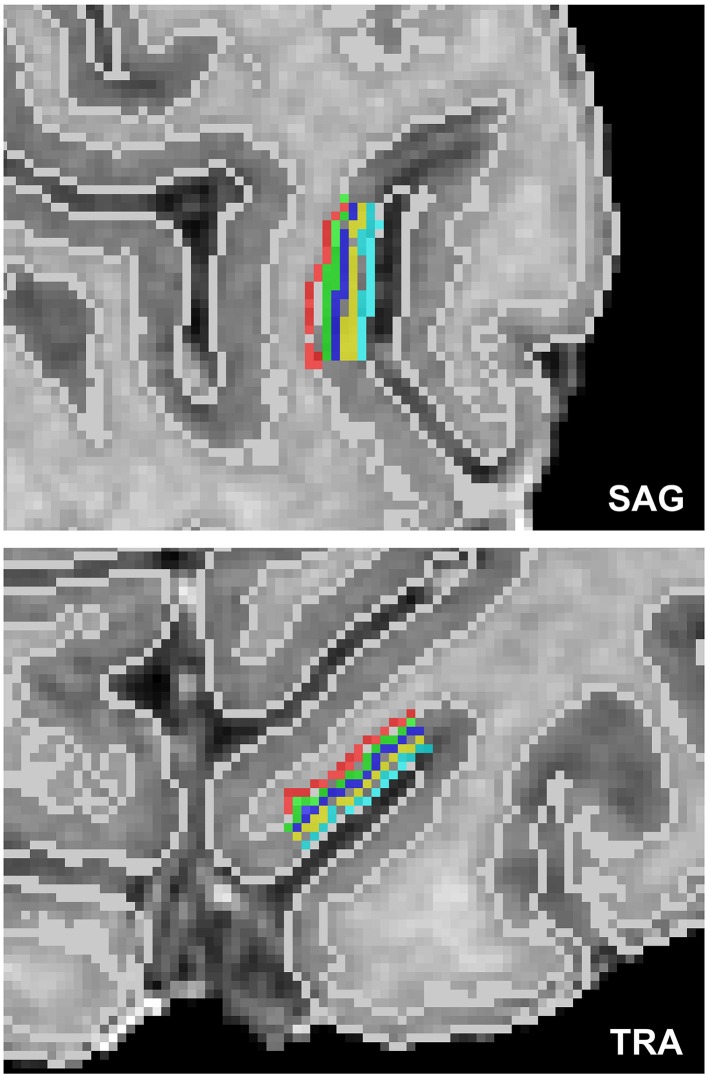
**Exemplary high resolution cortical grid sampling prescription**. Cortical depth dependent grids were prescribed at 10% (red), 30% (green), 50% (dark blue), 70% (yellow), and 90% (light blue) cortical depth as defined between the white matter–gray matter boundary and the gray matter–CSF boundary (white lines), as segmented based on the anatomical reference images.

For the cortical depth profile analysis, high resolution cortical depth grids were created similar to as described in De Martino et al. (2013) based on anatomical data after segmenting cortical gray and white matter and delineating the boundary between gray matter and cerebrospinal fluid (CSF). The automatic segmentation in BrainVoyager QX was used and refined manually in the regions of interest to ensure correct classification of white matter, gray matter and other tissue types. In each region of interest, a stack of five cortical grids were created starting from cortical thickness measurements (Jones et al., 2000). Grids were placed at regular intervals of cortical depth, and neighboring sampling points within a grid were regularly spaced. A single grid would sample 8 × 8 mm^2^ on a flat piece of cortex. A total of 10 regions of interest were defined within the foldings of the calcarine sulcus. Figure 1 displays an exemplary cortical depth sampling region of interest.

After the creation of relative cortical depth grids, the local cortex normal vectors (the vectors orthogonal to the cortical sheet) were approximated linearly by calculating the difference vector between the grid points at 10% cortical depth and the corresponding grid points at 90% cortical depth. The angle between these cortex normal vectors and the orientation of the partition directions of both 3D-GRASE 1 and 3D-GRASE 2 were calculated.

## Results

### Imaging point-spread function simulations and measurements

**Figure 2:**
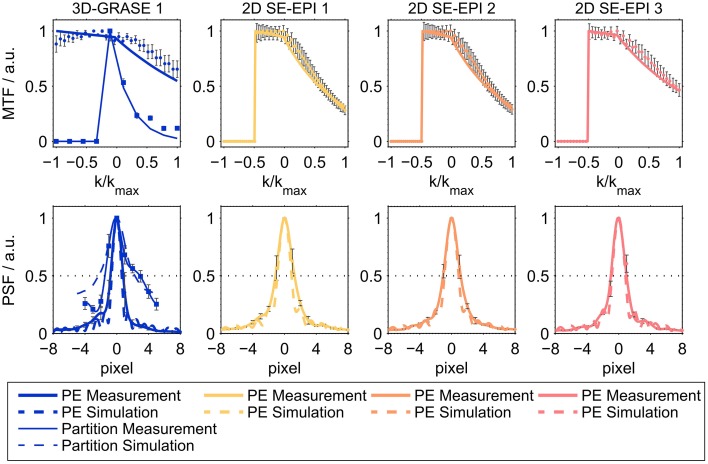
**Averaged modulation transfer functions (MTFs, top) and point-spread functions (PSFs, bottom)**. The top panel shows averaged MTFs in phase-encoding direction (thick lines) and partition encoding direction for 3D-GRASE sequences (thin lines). Solid lines represent averaged experimental results across all subjects, dashed lines represent simulations. The dimension of the horizontal axis in the top panel is relative k-space. In the bottom panel, the dimension of the horizontal axis is pixels in absolute numbers (cropped to center) for better comparison. Error bars indicate standard deviation across subjects.

Figure 2 shows the results of the simulations and the imaging PSF measurement for all acquisitions, demonstrating good agreement between experimental data and simulations for both the MTF (top) and the PSF (bottom). Prior to the creation of the MTFs, the raw data from the PSF measurements were coil-combined (sum-of-squares combination). The maximum of each readout line (i.e., the echo) was selected and averaged across the other imaging dimension (slices/partition direction for phase-encoding direction; phase-encoding direction for partition direction), and normalized in the range [0, 1]. Note the asymmetry in MTF with higher intensities in the echoes before than after the central spin-echo as expected from combined T_2_ and T_2_′ modulation. This signal characteristic is often neglected in over-simplified models assuming only a symmetric T^*^_2_ signal decay around the spin-echo.

Table 2 reports the mean across lines and subjects of the full width half maximum of the PSF (mean ± standard deviation). The FWHM of the partition direction in 3D-GRASE 1 was found to be 3.6 ± 0.5 pixels, which is about 56 and 63% higher than the FWHMs of the phase-encoding direction of 2D SE-EPI 1 and 2. Phase-encoding direction of 2D SE-EPI 3 and 3D-GRASE 1 acquisitions have smaller FWHMs due to the reduced number of phase-encoding steps. The experimental findings are in good agreement with the simulations. The agreement is enhanced when using the experimentally obtained volumetric T^*^_2_ values (right column) in the simulation rather than literature values for gray matter (middle column).

**Table 2:** **FWHM of the PSFs of the different acquisitions (mean ± standard deviation)**.

**Acquisition**	**Direction**	**FWHM (pixel)**		
		**Measurement**	Simulation GM T^*^_2_	Simulation Exp. T^*^_2_
3D-GRASE 1	PE	1.8 ± 0.3	1.2	1.3 ± 0.0
2D SE-EPI 1	PE	2.3 ± 0.2	2.0	2.3 ± 0.1
2D SE-EPI 2	PE	2.2 ± 0.1	2.0	2.3 ± 0.1
2D SE-EPI 3	PE	2.0 ± 0.1	1.8	2.0 ± 0.1
3D-GRASE 1	partition	3.6 ± 0.5	4.4	

**Figure 3:**
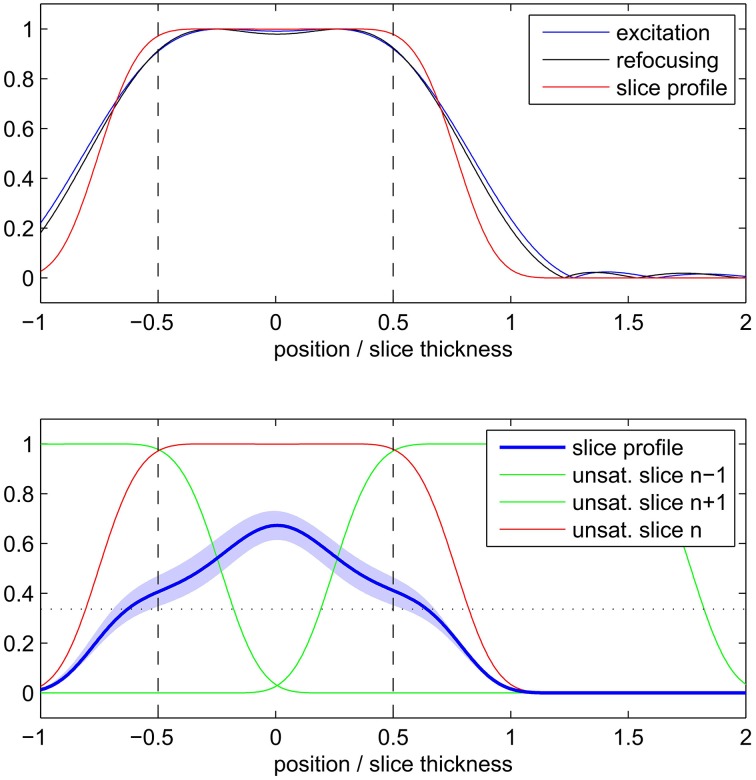
**Simulated 2D SE-EPI slice profile of a single slice (top) and with consideration of neighboring slices and saturation recovery (bottom)**. Profiles are displayed relative to nominal slice thickness.

The simulation results of the 2D SE-EPI slice profile are displayed in Figure 3. The FWHM (indicated by the vertical dashed line in the figure) was found to be 1.3 ± 0.2 times the nominal slice thickness (mean ± SD across different T_1_ values and saturation scenarios). Due to the wide saturation bands of neighboring slices, the effective slice profile resembles a triangle rather than the desired rectangle. When taking into account the saturation bands of neighboring slices, the integral of the attainable signal is reduced by 49 ± 5 percent compared to a single slice without T_1_ saturation effects and by 35 ± 5 percent compared to a single slice with T_1_ saturation effects.

### Temporal signal-to-noise ratio

**Figure 4:**
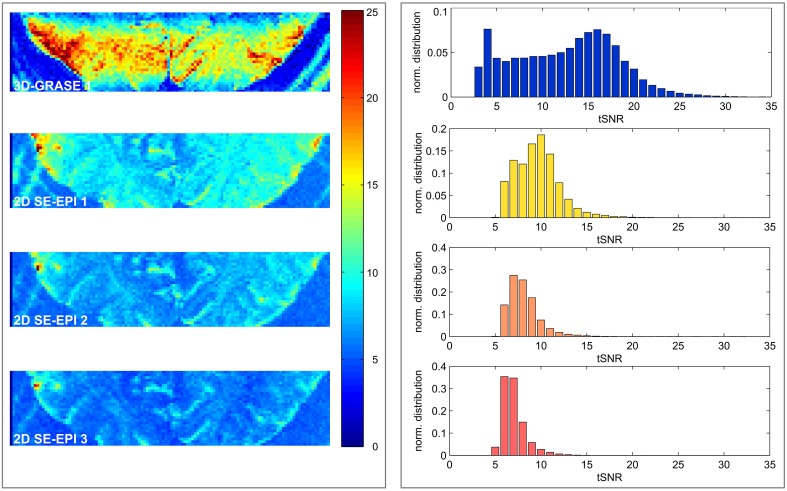
**Temporal signal-to-noise-ratio maps**. Maps of the five different acquisitions in a single subject **(left)** and corresponding histograms **(right)** are displayed. The 2D SE-EPI images are cropped to the same FoV as 3D-GRASE 1. All maps have the same color scale. Histograms display the tSNR distribution of the same in-plane FoV in all 3D-GRASE slices and 2D SE-EPI slices corresponding to 3D-GRASE 1.

Figure 4 displays central slices of the tSNR maps and full volume tSNR histograms. The 2D SE-EPI data were cropped to the same in-plane FoV as the 3D-GRASE 1 acquisition (disregarding increased image distortions in 2D SE-EPI). The images and histograms show that the tSNR is substantially higher in 3D-GRASE than in all 2D SE-EPI acquisitions. 3D-GRASE 2 yields similar tSNR as 3D-GRASE 1 (not shown because of its different slice orientation). The 2D SE-EPI 1, 2, and 3 [short TE (*R* = 2), long TE (*R* = 2), long TE (*R* = 3)] yield tSNR in descending order as expected due to T_2_ relaxation, undersampling (*R*), and noise amplification (g-factor) from higher acceleration. Similar results were found for all other subjects (not shown).

### Visual activation

**Figure 5:**
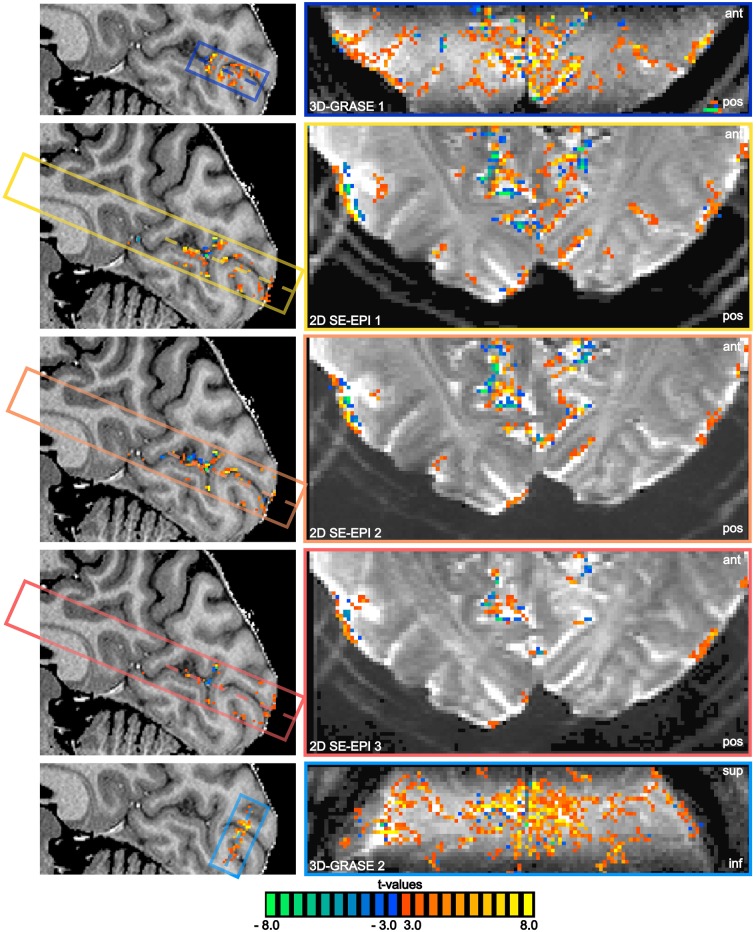
**BOLD responses overlaid on anatomical reference and averaged functional slices**. Mid-sagittal slices of the reference anatomical scan **(left)** and central functional slices **(right)** are shown, as indicated by the dashed lines. Note the oblique coronal orientation of 3D-GRASE 2. The same statistical threshold was applied to all acquisitions (*t* > 3.0, *p* < 0.003 two-sided *t*-test on single voxel level, cluster threshold of 4 voxels). The position of the sagittal cut is shown on a transversal slice in the bottom left corner. The extent and orientation of the functional slices as well as the displayed slice position (dashed lines) are indicated by the colored boxes in the sagittal images.

All functional acquisitions elicited significant responses to the visual stimulation in and around the calcarine sulcus. Figure 5 displays results of the functional experiment overlaid on both an anatomical reference (sagittal cut, left), and on averaged functional slices (single central slice). The same statistical threshold was chosen for all acquisitions, and no multiple comparison correction was used in order not to favor the reduced FoV acquisitions. Note the absence of activated voxels in white matter regions in all acquisitions. BOLD sensitivity is significantly lower in the 2D SE-EPI acquisitions as a consequence of the reduced tSNR (see Figure 4).

**Figure 6:**
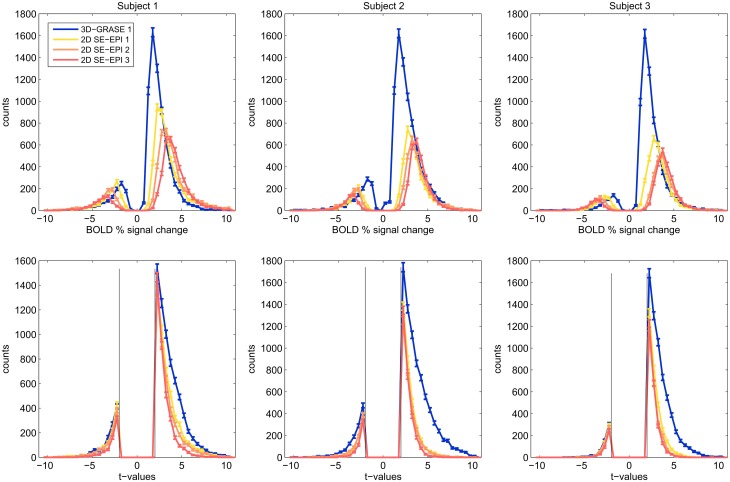
**Histograms of significant voxels**. Counts of BOLD signal changes (top) and *t*-values are shown for voxels, which responded significantly to the stimulation (*t* > 2.0, *p* < 0.046).

Figure 6 displays histograms of the BOLD signal changes (top) and *t*-values (bottom) for all three subjects. For each acquisition, voxels were included in the analysis if they responded significantly (*p* < 0.05 uncorrected). Only the mutually covered region was considered. Border voxels were discarded to exclude artifacts from motion or slow scanner drift. The histograms reveal that in each subject, smaller BOLD signal changes surpass the significance threshold using 3D-GRASE, indicative of smaller variability in the responses. In consequence, more active voxels are detected in 3D-GRASE 1 than in any of the 2D SE-EPI acquisitions (see Figure 6 bottom).

### Cortical depth profiles

**Figure 7:**
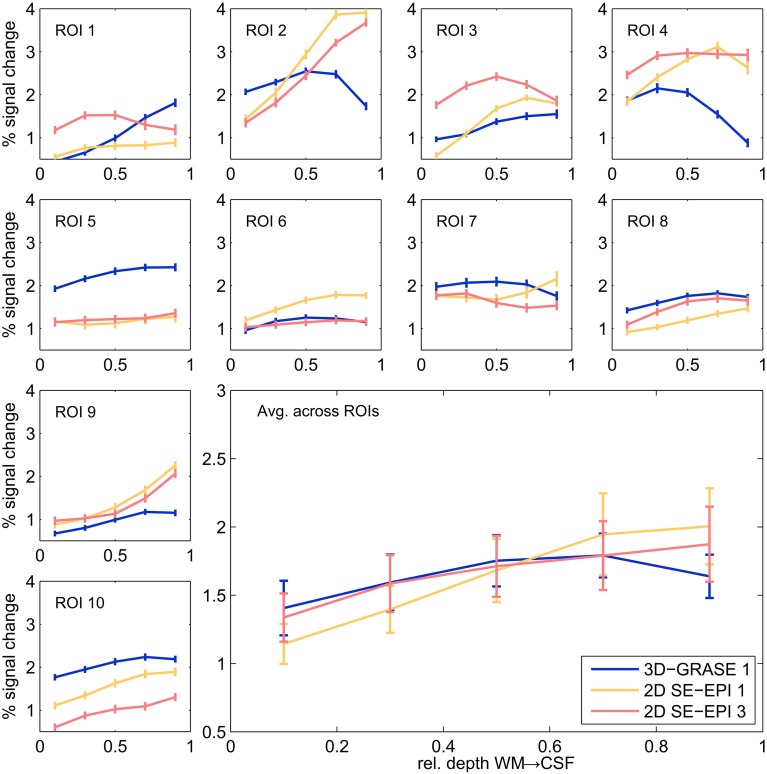
**Cortical depth profiles in individual regions**. Single regions (small graphs) and their average (large graph, bottom right) of 3D-GRASE 1 (blue), 2D SE-EPI 1 (yellow), and 2D SE-EPI 3 (pink). Error bars represent standard errors across grid points (individual ROI graphs) and across regions (average graph).

Figure 7 displays cortical depth profiles from individual regions and their average from 3D-GRASE 1, 2D SE-EPI 1, and 2D SE-EPI 3. 2D SE-EPI 2 was similar to 2D SE-EPI 1 and 2D-SE-EPI 3 (not shown for clarity). Grid points were included in the spatial average, if at least one of the three displayed acquisitions elicited significant responses (*p* < 0.05, uncorrected) in at least one of the five cortical depths. The responses measured with these protocols are similar showing BOLD signal changes mostly between 1 and 3%. In both 2D SE-EPI and 3D-GRASE the BOLD signal change is significantly higher at 50% cortical depth than at 10% (two-sided Wilcoxon signed-rank test, *p* < 0.02, uncorrected). The 2D SE-EPI acquisitions increase further toward the pial surface (signal change is different between 50 and 90% at *p* < 0.02, two-sided Wilcoxon signed-rank test, for 2D SE-EPI 1 and 2, not significant for 2D SE-EPI 3), whereas the 3D-GRASE profile shows a trend to decrease toward the surface.

**Figure 8:**
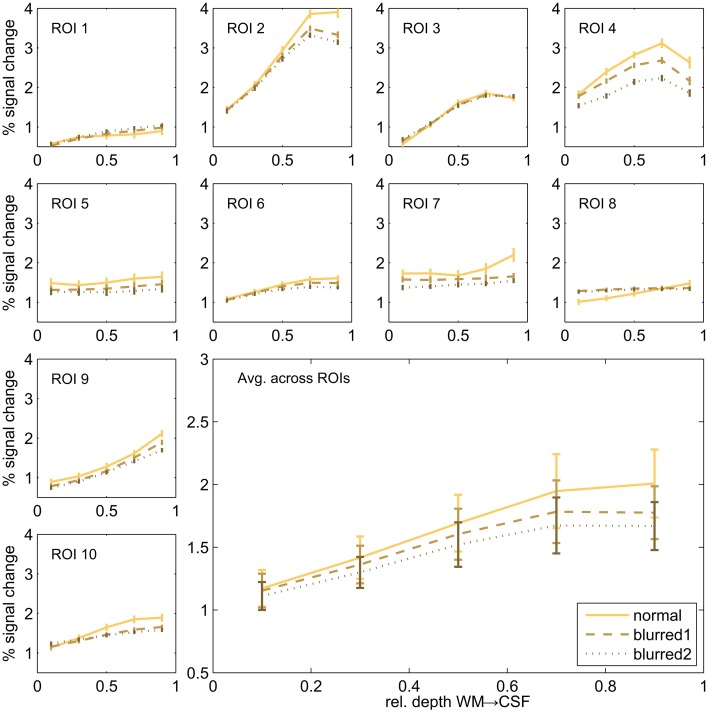
**Cortical depth profiles in individual regions**. Single regions (small graphs) and their average (large graph, bottom right) of 2D SE-EPI 1. Original data is compared to blurred data of two different blurring kernel sizes.

In order to assess the potential effect of the imaging PSF on the cortical depth profiles dependent profiles, we repeated the cortical depth profile analysis after smoothing the 2D SE-EPI 1 data using an anisotropic Gaussian kernel. We used two different sizes of the smoothing kernel (FWHM of 2.4 and 4.7 pixels) in slice direction (no additional smoothing in other directions) a range that includes the estimated FWHM of 3D-GRASE 1 in the partition direction. The comparison of normal and blurred data is shown in Figure 8. The noted increase toward the pial surface in 2D SE EPI remains despite the smoothing. All profiles differ significantly between 10 and 90% cortical depth (two-sided Wilcoxon signed-rank test, *p* < 0.002, uncorrected).

**Figure 9:**
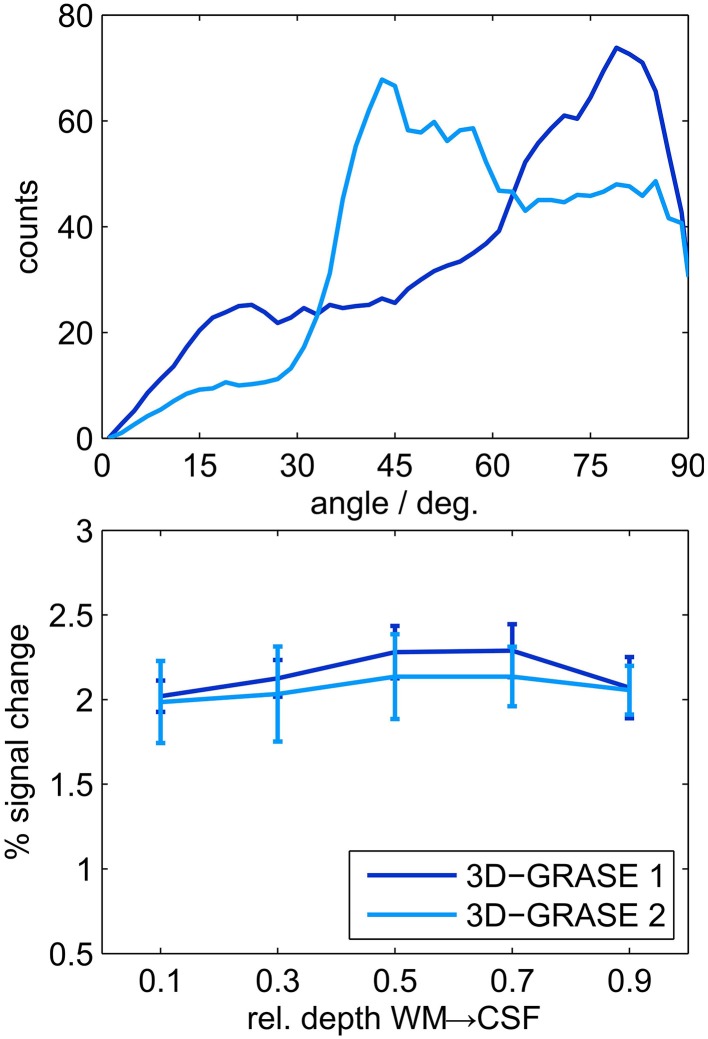
**Comparison of 3D-GRASE 1 and 3D-GRASE 2. Top:** Histograms of angle between cortex normal and partition encoding direction of both, 3D-GRASE 1 (dark blue) and 3D-GRASE 2 (light blue), in degrees. **Bottom:** Mean cortical depth profiles. Error bars represent standard errors across regions.

We also evaluated the difference between 3D-GRASE 1 and the orthogonally oriented 3D-GRASE 2 in four of the individual regions which were sufficiently covered by both acquisitions (ROIs 2, 5, 7, and 10 in Figure 7). Histograms of the angle (limited between 0 and 90°) between the cortex normal vectors and the partition directions of both 3D-GRASE 1 and 2, are presented at the top of the Figure 9. The averages of the cortical depth profiles in these regions are displayed at the bottom in the same figure. Despite the difference in acquisition orientation the depth dependent functional profiles are similar to one another, and are similar to the 3D-GRASE 1 profile in Figure 9 although only a subset of ROIs was used.

## Discussion

In this study, we compared 3D-GRASE to 2D SE-EPI acquisitions in a high spatial resolution BOLD fMRI experiment at 7 T. Simulations, PSF measurements and a simple visual activation paradigm with robust functional responses were used to assess differences with regard to voxel blurring, functional sensitivity, and functional (BOLD) specificity as indicated by cortical depth dependent profiles. Our findings suggest that the anisotropic blurring in 3D-GRASE is higher than in 2D SE-EPI. Further, 3D-GRASE yields a more sensitive BOLD signal and less macrovascular weighting as indicated by the smaller superficial effect in the cortical depth profiles. Based on these findings, we conclude that with the used experimental set-up 3D-GRASE yields valuable advantages over 2D SE-EPI for high-resolution fMRI applications provided the more limited coverage is sufficient to the experimental setup. The ability to discriminate small, differential signals at a length-scale on the order of 1 mm depends on the product of tSNR and effect-size (Murphy et al., 2007), which is proportional to the contrast-to-noise ratio (CNR). The measured effect-size (i.e., difference in BOLD signal) depends on the image blurring, and hence depends on the orientation of the structure with respect to the anisotropic image blurring. It can be shown that, for the protocols employed here, the superior tSNR of 3D-GRASE outweighs detrimental effects of the stronger image blurring, and that the CNR will be below that of 2D SE-EPI only if the investigated structure is closely aligned (~0°) with the partition direction of 3D-GRASE.

Our results are in general agreement with previous studies investigating 3D-GRASE (De Martino et al., 2013) or 2D SE-EPI (Goense and Logothetis, 2006; Harel et al., 2006; Jin and Kim, 2008), independently. The different protocols we used followed practical considerations and were performed similarly to previously published studies while also matching parameters such as resolution and echo time. Compared to previous studies, the imaging FoV of the 3D-GRASE acquisitions was reduced to account for the slower switching of the body gradient coil (SC72) that was used here.

Inner-volume selection was previously proposed to reduce the echo train length in 2D SE-EPI using spatially orthogonal refocusing pulses to acquire a single slice (Yacoub et al., 2007, 2008). Multi-slice acquisitions with this approach are sub-optimal because of saturation effects. Outer-volume suppression (OVS, Heidemann et al., 2012) and 2D spatially selective RF pulses (Pauly et al., 1989; Finsterbusch, 2010, 2013) have also been proposed. However, magnetization transfer effects in OVS would reduce the tSNR of 2D SE-EPI further, while additional RF-pulses would tighten the SAR constraints (Pfeuffer et al., 2002; Wargo and Gore, 2013) leading to a reduced number of slices. The use of segmented acquisitions would require techniques to mitigate the effects of subject motion (e.g., the use of bite-bars or advanced image reconstruction techniques) and would be significantly less time efficient. Finally, a surface receive coil with an even more limited FoV (Koning et al., 2013) was not available to us and may represent an alternative way to achieve zoomed imaging in 2D multi-slice imaging.

### Imaging point-spread function

3D-GRASE was found to exhibit a wider PSF in partition direction than the 2D SE-EPI acquisitions in phase-encoding direction or slice direction (as expected from simulations). The blurring in the phase-encoding direction of 3D-GRASE was minimal because of the short EPI echo train duration due to the reduced FoV.

The PSF was assessed based on non-phase-encoded data from the entire FoV. Two concomitant sources of possible confounds should be taken into consideration. First, different spatial locations have different PSF properties due to the relaxation properties of their underlying anatomical tissue. Since T_2_ of white matter and gray matter is almost identical at 7 T (Yacoub et al., 2003; Deistung et al., 2008; Cox and Gowland, 2010) and T^*^_2_ of white matter is only slightly shorter (Peters et al., 2007; Deistung et al., 2008), one can expect this influence to be small. CSF, which has a much longer T_2_, occupies only a small volume fraction in the chosen FoV and is partly saturated due to its long T_1_ (T_1_ ~ TR). Other tissue compartments, which have much shorter T_2_, contribute relatively little to the signal at TE. It can therefore be expected that the presence of various tissue types does not significantly affect the PSF estimation. Second, static field inhomogeneities across the FoV lead to an overestimation of the global PSF by a broadening of the measured signal. Local off-resonances shift the voxel-by-voxel peak along the phase-encoding direction. The sum of the shifted peaks is broader than an individual one would be. Also, the effective volumetric T^*^_2_ is shortened. Either of these effects (tissue or field specific inhomogeneities) would lead to an overestimation or larger PSF. As such, our measurements can be regarded as a worst-case approximation of the local blurring. The simulations using gray matter T^*^_2_, on the other hand, reflect the ideal case and can be seen as a lower bound of the local blurring, not subject to either experimental imperfections affecting the local blurring or the global overestimation affecting only our measurements. The reasonable agreement of these numbers suggests that the detrimental effects of the global estimation are limited. An alternative approach, circumventing these possible confounds, would be to employ an acquisition scheme similar to the one by (Zeng and Constable, 2002; Chung et al., 2011), which was used for distortion correction in EPI images. In this study, we chose an approach that did not depend on image reconstruction, which is desirable when comparing different imaging pulse sequences.

This work focused on imaging related effects on the PSF and we did not consider the additional effect of the BOLD related spatial spread of the signal (i.e., the functional PSF), both of which need to be accounted for in determining the ultimate resolution of the fMRI signals. The latter is normally measured by considering differential paradigms with a known spatial distribution of responses and comparing it to the measured spatial distribution (Shmuel et al., 2007). In light of the results presented here, it is worth noting that a wider spatial imaging PSF will not cancel out high spatial frequency information in differential fMRI paradigms. Rather, when adjacent areas respond to different functional conditions, a wider spatial imaging PSF has a similar effect as a wider functional PSF and results in a reduction of CNR between the functional conditions (Yacoub et al., 2008). Thus, the combination of a differential fMRI paradigm, along with a high functional CNR with reduced contamination from pial veins, explain why columnar or cortical depth dependent fMRI analyses (Zimmermann et al., 2011; Olman et al., 2012; De Martino et al., 2013), with blurring levels (in 3D GRASE acquisitions) comparable to the data presented here, are possible.

### tSNR and BOLD sensitivity

The sensitivity to detect small, significant BOLD changes was found to be clearly superior in 3D-GRASE in this study (Figures 5, 6). This is a straight-forward consequence of the higher tSNR (Figure 4). The tSNR advantage of 3D-GRASE is likely to stem from two sources: First, being a 3D sequence 3D-GRASE is more SNR efficient because of the longer effective sampling time per volume (Edelstein et al., 1986). This is particularly relevant since such high-resolution fMRI typically operates in the thermally dominated noise regime (Triantafyllou et al., 2005), in which tSNR scales linearly with SNR. Second, the inner volume selection in 3D-GRASE obviated the need for parallel imaging in 3D-GRASE, whereas it was necessary in 2D SE-EPI causing a loss in static SNR and hence tSNR by under-sampling and g-factor penalty. Additionally, although the FWHM of the 2D SE-EPI acquisitions was wider in the slice direction than expected (1.3 ± 0.2 times the nominal slice thickness) the large overlap of neighboring slices in the multi-slice 2D acquisitions reduces the available magnetization because of saturation effects (Figure 3). Improved slice profiles could therefore improve both tSNR and point-spread.

The origin of the greater sensitivity of the 3D-GRASE sequence seems not to originate from differences in functional contrast (i.e., 3D-GRASE did not exhibit greater response amplitudes, as seen in the functional response profiles, which is also consistent with less contribution from pial vessels), but rather the higher sensitivity seems to originate from a more robust signal (i.e., less noise). The higher sensitivity permitted 3D-GRASE to detect more subtle BOLD signal changes than 2D SE-EPI, while larger responses were detected about equally.

### Contrast mechanisms and functional specificity

All acquisitions employed in this study elicit primarily T_2_ weighted contrast, with varying contributions from other mechanisms. As such, the bulk of the detected BOLD signal changes have absolute amplitudes below five percent, in agreement with the literature (Yacoub et al., 2005), however, it should be noted that differences in tasks, acquisition parameters (e.g., echo time, Koopmans et al., 2011), or sampling strategies may all result in differences in the observed functional signal changes. Contributions from T^*^_2_ (T_2_′) are not negligible due to the duration of the EPI echo trains, which was on the order of magnitude of the T^*^_2_ of the brain tissue. The T^*^_2_ contribution is expected to be higher in the 2D SE-EPI acquisitions than in 3D-GRASE because of the longer EPI echo trains and in-plane partial Fourier acquisitions, as discussed in the section on the image PSF. The later (non-center) partitions in the 3D-GRASE echo-train are sampled at a later effective TE than the nominal TE, which was matched with the TE of 2D SE-EPI 1. Therefore, the T_2_ contribution is higher in 3D-GRASE because later partitions experience more T_2_ weighting and additional T_2_ weighted stimulated echo weighting than the center partition (Goerke et al., 2007). In addition, blood vessels have a particularly sharp spatial profile (compared to smoother gray or white matter structures), which manifests itself in the k-space regions corresponding to high spatial frequencies. High k-space lines of an EPI readout, in turn, experience the strongest T^*^_2_ weighting. Note that, in reduced FoV acquisitions, the same effect takes place. However, given the shorter EPI readout times, the high k-space lines are acquired closer to the spin-echo, thus reducing the effect size.

Finally, more diffusion weighting is aggregated along the readout train of 3D-GRASE due to the readout and crusher gradients. Moderate diffusion weighting has been shown to suppress macrovascular signals (Lee et al., 1999; Duong et al., 2003; Yacoub et al., 2003).

In consequence of the aforementioned differences in contrast between 3D-GRASE and 2D SE-EPI, the cortical depth profiles differ such that 2D SE-EPI exhibits an increase of the signal change toward the pial surface, whereas 3D-GRASE remains mostly flat. Such increasing cortical depth profiles have previously been associated with reduced functional specificity in SE-EPI (Goense and Logothetis, 2006) and GE-EPI (Polimeni et al., 2010; De Martino et al., 2013), because they reflect T^*^_2_ induced weighting of macrovasculature. We follow this line of argument and conclude that under the experimental conditions employed here, 3D-GRASE is less biased by large superficial effects as indicated by the layer profiles (Figure 7).

Our imaging PSF results raise the question of whether the observed difference in layer profiles is a mere consequence of the stronger blurring in the partition direction of 3D-GRASE. We show that this is not the case in two ways. First, we smoothed the 2D SE-EPI 1 data with anisotropic Gaussian filters exceeding the width of the PSF of 3D-GRASE along the partition direction. The characteristics of cortical depth profiles, and more specifically the increase toward the pial surface, are preserved (Figure 8). Second, when looking at the angle between the partition encoding direction and the direction of the cortical depth (Figure 9 top), on average there is not a bias of alignment between the partition direction and the grid direction (i.e., smaller angles between the directions). The curvature of the cortex prevents this from occurring and ultimately protects the cortical depth measures from the partition blurring of both 3D-GRASE 1 and 3D-GRASE 2, despite their orthogonal prescription. Further, the differences in angle distributions between the two acquisitions did not even elicit any significant difference in the layer profiles (Figure 9).

### Conflict of interest statement

The authors declare that the research was conducted in the absence of any commercial or financial relationships that could be construed as a potential conflict of interest.
